# Supplementary material for: Phosphorus stress induces the synthesis of novel glycolipids in Pseudomonas aeruginosa that confer protection against a last-resort antibiotic
Source: ISME J. 2021 May 24;15(11):3303–14. doi: 10.1038/s41396-021-01008-7 (PMC8528852; doi:10.1038/s41396-021-01008-7)
Supplement: Supplementary file 1 — supplementary figure 1 [file 41396_2021_1008_MOESM1_ESM.docx]

**Supplementary Figures and Tables**

**Suppl. Figure S1** Growth of the *P. aeruginosa* mutants in the minimal medium under Pi repleted and Pi depleted conditions.

**Suppl. Table S1** Proteomic analysis of differentially expressed proteins in the wild-type *P. aeruginosa* PAO1 in response to different Pi levels (1 mM versus 50 µM).

**Suppl. Table S2** Protein BLAST identification of locus tags homologous to *agt1* and *agt2* glycolipid synthesis genes in all genome-sequenced *P. aeruginosa* strains at the JGI IMG database.

**Suppl. Table S3** Proteomic analysis of wild type *P. aeruginosa* PAO1 versus the Δ*plcP* mutant grown at 50 µM Pi.

**Suppl. Table S4** Whole genome mapping to *Pseudomonas aeruginosa* PAO1 of metatranscriptomic datasets (SRX5145605, SRX5145606, SRR6833349) from sputum samples taken from cystic fibrosis patients and a patient with chronic wound infection (SRR6833340). **S4A** (SRX5145605)**, S4B** (SRX5145606), **S4C** (SRR6833349), **S4D** (SRR6833340)**.** RPKM, reads per kilobase of transcript per million mapped reads; FPKM, fragments per kilobase of transcript per million mapped reads.


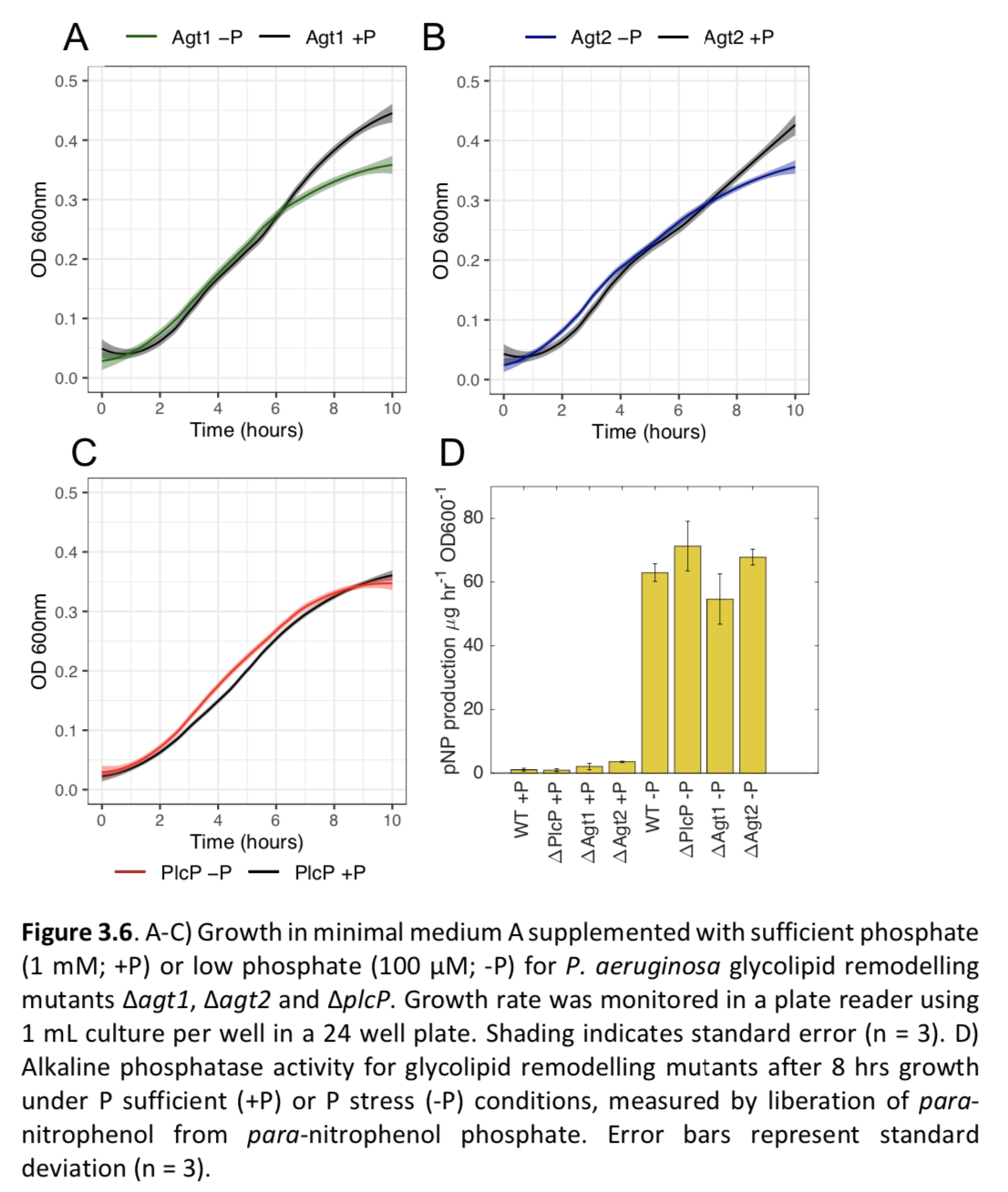


**Supplementary Figure 1**, A-C) Growth of the *P. aeruginosa* mutants in the minimal medium supplemented with sufficient phosphate (1 mM, “+P”) or low phosphate (100 µM, “-P”). Growth was monitored in a plate reader using 1 mL of culture per well in a 24-well plate. Shading indicates standard error (n=3). D) Alkaline phosphatase activity assays for mutants after 8 hrs growth under +P and -P conditions. Error bars represent standard deviation (n=3).
