# Supplementary material for: Phosphorus stress induces the synthesis of novel glycolipids in Pseudomonas aeruginosa that confer protection against a last-resort antibiotic
Source: ISME J. 2021 May 24;15(11):3303–14. doi: 10.1038/s41396-021-01008-7 (PMC8528852; doi:10.1038/s41396-021-01008-7)
Supplement: Supplementary file 2 — supplementary table 1 [file 41396_2021_1008_MOESM2_ESM.docx]

**Supplementary table 1** Proteomic analysis of differentially expressed proteins in the wild-type *P. aeruginosa* PAO1 in response to different phosphate (Pi) levels (1 mM versus 50 µM). Locus tags from the *Pseudomonas* genome reference database are listed alongside known or predicted protein functions. Log2 transformation of fold change values show the differences between high and low Pi *P. aeruginosa* cultures, and are the mean of 3 replicates. All proteins displayed have a log2(Fold Change) of ≥2 and all were considered significant with a false detection rate of <0.05 (FDR<0.05). Proteins that are more represented in low Pi culture are indicated with negative values.

| **Protein function** | **Locus Tag** | **log2(Fold Change)** |
| --- | --- | --- |
| Non-hemolytic phospholipase C (PlcN) | PA3319 | -10.20673752 |
| Extracelullar DNA degradation protein (EddA) | PA3910 | -9.755251567 |
| Uncharacterized protein | PA2635 | -9.698588053 |
| Phenazine biosynthesis protein (PhzE) | PA4214 | -8.654287974 |
| Probable glutamine amidotransferase | PA3459 | -8.394351323 |
| Glycerophosphoryl diester phosphodiesterase (GlpQ) | PA0347 | -8.350893021 |
| Uncharacterized protein | PA5061 | -8.117766062 |
| Polyphosphate:ADP phosphotransferase | PA2428 | -7.436549505 |
| Rhamnosyltransferase chain B | PA3478 | -7.369407018 |
| Probable non-ribosomal peptide synthetase | PA4078 | -7.135940552 |
| Extracelullar DNA degradation protein (EddB) | PA3909 | -7.1205616 |
| Probable two-component response regulator | PA2881 | -6.924730937 |
| Uncharacterized protein | PA0690 | -6.878400167 |
| Probable carbamoyl transferase | PA2069 | -6.420543671 |
| Pyruvate dehydrogenase (cytochrome) | PA5297 | -6.189308167 |
| 3-(3-hydroxydecanoyloxy)decanoate synthase | PA3479 | -6.098758062 |
| Probable bacterioferritin | PA4880 | -6.079444885 |
| Uncharacterized protein | PA0698 | -5.714225133 |
| Alkaline phosphatase H | PA3296 | -5.619827906 |
| Rhamnosyltransferase 2 | PA1130 | -5.496246974 |
| Phenazine biosynthesis protein (PhzD2) | PA1902 | -5.485527039 |
| Uncharacterized protein | PA2171 | -5.434581121 |
| Probable acetyltransferase | PA3368 | -5.278530757 |
| Poly(3-hydroxyalkanoic acid) synthase 2 | PA5058 | -5.249589284 |
| Anthranilate--CoA ligase | PA0996 | -5.135194778 |
| Phospho-2-dehydro-3-deoxyheptonate aldolase | PA4212 | -5.017737071 |
| Transport protein (ExbB2) | PA0693 | -4.948032379 |
| Uncharacterized protein | PA3374 | -4.706494013 |
| Uncharacterized protein | PA3219 | -4.624661764 |
| Probable type II secretion system protein | PA0685 | -4.622166316 |
| Poly(3-hydroxyalkanoic acid) depolymerase | PA5057 | -4.482566198 |
| Uncharacterized protein | PA1606 | -4.477079391 |
| UDP-glucose 6-dehydrogenase | PA2022 | -4.273289998 |
| Uncharacterized protein | PA2804 | -4.227488836 |
| Uncharacterized protein | PA0692 | -4.22598203 |
| Uncharacterized protein | PA1324 | -4.198731105 |
| Probable glycosyl transferase | PA0842 | -4.078671137 |
| Methyl-accepting chemotaxis protein (CtpH) | PA2561 | -4.039987564 |
| Uncharacterized protein | PA0695 | -3.896328608 |
| Phosphonates import ATP-binding protein (PhnC2) | PA3384 | -3.790336609 |
| Porin P | PA3279 | -3.78040123 |
| Membrane protein component of ABC phosphate transporter | PA5368 | -3.768856684 |
| Biotin synthase | PA0500 | -3.715056101 |
| Probable two-component sensor | PA2882 | -3.714621862 |
| Uncharacterized protein | PA2433 | -3.680545171 |
| Phosphate regulon transcriptional regulatory protein (PhoB) | PA5360 | -3.665147781 |
| Probable transcriptional regulator | PA0701 | -3.653577805 |
| Antimetabolite l-2-Amino-4-Methoxy-trans-3-Butenoic Acid (AMB) synthesis (AmbE) | PA2302 | -3.63861084 |
| Uncharacterized protein | PA2167 | -3.637429555 |
| Peptidylprolyl isomerase | PA0699 | -3.605301539 |
| Probable acetyltransferase | PA3460 | -3.552387238 |
| Probable short-chain dehydrogenase | PA2918 | -3.534699122 |
| Uncharacterized protein | PA0460 | -3.524803162 |
| Phenazine/pyocyanine biosynthesis protein (PhzF) | PA4215 | -3.515024185 |
| Acyl-homoserine-lactone synthase (LasI) | PA1432 | -3.477566401 |
| Anthranilate synthase component 1, pyocyanine specific (ASI) | PA1001 | -3.471937815 |
| Trans-aconitate 2-methyltransferase | PA2564 | -3.468770981 |
| Osmotically inducible protein (OsmC) | PA0059 | -3.428434372 |
| Uncharacterized protein | PA4738 | -3.427829107 |
| Phosphate transport system permease protein (PstA) | PA5367 | -3.409196218 |
| Lipotoxon F (LptF) | PA3692 | -3.365186056 |
| HxcT pseudopilin | PA0681 | -3.364541372 |
| Uncharacterized protein | PA4739 | -3.36428388 |
| Uncharacterized protein | PA3461 | -3.343550364 |
| (R)-3-hydroxydecanoyl-ACP:CoA transacylase (Quinolone sensitivity protein) | PA0730 | -3.319576899 |
| Uncharacterized protein | PA3819 | -3.310759226 |
| Probable Resistance-Nodulation-Cell Division (RND) efflux transporter | PA4207 | -3.309268316 |
| Uncharacterized protein | PA2915 | -3.25749588 |
| ECF sigma factor (VreI) | PA0675 | -3.25052007 |
| Probable transcriptional regulator | PA0275 | -3.245417277 |
| 2-heptyl-4(1H)-quinolone synthase subunit (PqsC) | PA0998 | -3.231575012 |
| Uncharacterized protein | PA4139 | -3.210337321 |
| Uncharacterized protein | PA3691 | -3.20965449 |
| Uncharacterized protein | PA1216 | -3.198550542 |
| Periplasmic gluconolactonase (PpgL) | PA4204 | -3.192895253 |
| Phenazine biosynthesis protein PhzA1 | PA4210 | -3.161492666 |
| Uncharacterized protein | PA4884 | -3.11555926 |
| Probable aldehyde dehydrogenase | PA2378 | -3.089309692 |
| Probable trehalose synthase | PA2152 | -3.038731257 |
| Uncharacterized protein | PA0696 | -3.033399582 |
| Uncharacterized protein | PA2803 | -3.010194143 |
| Putative quercetin 2,3-dioxygenase (Putative quercetinase) | PA3240 | -3.00465711 |
| Methyl-accepting chemotaxis protein (CtpL) | PA4844 | -3.001227697 |
| Binding protein component of ABC phosphonate transporter | PA3383 | -2.984984716 |
| Uncharacterized protein | PA5299 | -2.971493403 |
| Phosphate import ATP-binding protein (PstB) | PA5366 | -2.940657298 |
| Uncharacterized protein | PA3844 | -2.935982386 |
| Phenazine-1-carboxylate N-methyltransferase | PA4209 | -2.930987676 |
| Uncharacterized protein | PA1323 | -2.913213094 |
| Probable transcriptional regulator | PA3381 | -2.907595317 |
| Antimetabolite l-2-Amino-4-Methoxy-trans-3-Butenoic Acid (AMB) synthesis (AmbB) | PA2305 | -2.888827642 |
| Antimetabolite l-2-Amino-4-Methoxy-trans-3-Butenoic Acid (AMB) synthesis (AmbD) | PA2303 | -2.874830246 |
| Uncharacterized protein | PA3572 | -2.827032725 |
| Antimetabolite l-2-Amino-4-Methoxy-trans-3-Butenoic Acid (AMB) synthesis (AmbC) | PA2304 | -2.79532814 |
| Phospholipase C (PlcB) | PA0026 | -2.792703629 |
| Uncharacterized protein | PA2771 | -2.775707881 |
| Uncharacterized protein | PA3734 | -2.775616328 |
| Probable glycosyl hydrolase | PA2160 | -2.72849528 |
| 5-methyltetrahydropteroyltriglutamate--homocysteine methyltransferase | PA1927 | -2.690607707 |
| Chitinase | PA2300 | -2.678885142 |
| Catalase HPII | PA2147 | -2.6479702 |
| Uncharacterized protein | PA3846 | -2.640010198 |
| Putative zinc metalloprotease | PA3649 | -2.612800598 |
| Porin O | PA3280 | -2.550654093 |
| Sigma factor regulator (VreR) | PA0676 | -2.514614105 |
| Serine protease | PA1327 | -2.498445511 |
| Uncharacterized protein | PA3705 | -2.488183975 |
| Osmotically inducible lipoprotein (OsmE) | PA4876 | -2.47485288 |
| Uncharacterized protein | PA0697 | -2.450276693 |
| Protease (PfpI) | PA0355 | -2.429958979 |
| Alpha-1,4-glucan:maltose-1-phosphate maltosyltransferase | PA2151 | -2.419034958 |
| Uncharacterized protein | PA5446 | -2.347930272 |
| Adenosylhomocysteinase | PA0432 | -2.347044627 |
| Phosphate-binding protein (PstS) | PA5369 | -2.344860077 |
| Probable hydrolase | PA2067 | -2.336758296 |
| Aconitate hydratase A (ACN) | PA1562 | -2.323865255 |
| Uncharacterized protein | PA4993 | -2.306030273 |
| Uncharacterized protein | PA4205 | -2.290850957 |
| Phosphate-specific transport system accessory protein PhoU homolog | PA5365 | -2.276836395 |
| Probable metal-transporting P-type ATPase | PA3690 | -2.253185908 |
| Probable pyridoxamine 5'-phosphate oxidase | PA4216 | -2.240835826 |
| GTP 3',8-cyclase 2 | PA1505 | -2.193239848 |
| Protease (LasA) | PA1871 | -2.185289383 |
| Phosphate regulon sensor protein (PhoR) | PA5361 | -2.128717422 |
| 2,4-dienoyl-CoA reductase (FadH1) | PA3092 | -2.091956457 |
| Uncharacterized protein | PA0557 | -2.083759308 |
| Probable type II secretion system protein | PA0687 | -2.080123266 |
| Chloroperoxidase | PA2717 | -2.076198578 |
| Probable lipid kinase YegS-like | PA3023 | -2.0685908 |
| Probable Resistance-Nodulation-Cell Division (RND) efflux membrane fusion protein | PA4206 | -2.033835729 |
| Uncharacterized protein | PA2927 | -2.013376236 |
| Catalase | PA4236 | -2.008307139 |
| Putative imidazole glycerol phosphate synthase subunit (hisF2) | PA3151 | 1.974990209 |
| Uncharacterized protein | PA1513 | 2.102914174 |
| Ferric enterobactin receptor (PirA) | PA0931 | 2.332770665 |
| Probable antioxidant protein | PA3450 | 2.342319489 |
| Probable ATP-binding component of ABC transporter | PA2408 | 2.393850327 |
| Uncharacterized protein | PA5137 | 2.402357101 |
| Probable hydrolase | PA0562 | 2.449858348 |
| Probable binding protein component of ABC transporter | PA0602 | 2.498491923 |
| Probable MFS dicarboxylate transporter | PA5530 | 2.516822179 |
| Negative regulator of type III secretion (ExsD) | PA1714 | 2.575972239 |
| Regulator of liu genes | PA2016 | 2.58658282 |
| Uncharacterized protein | PA1746 | 2.639357885 |
| Probable amino acid binding protein | PA3865 | 2.641301473 |
| Uncharacterized protein | PA1043 | 2.665437698 |
| Uncharacterized protein | PA4132 | 2.67109553 |
| Probable binding protein component of ABC transporter | PA2204 | 2.726812363 |
| High-affinity branched-chain amino acid transport system permease protein (BraE) | PA1072 | 2.792919159 |
| Probable transcriptional regulator | PA0942 | 2.803567886 |
| Heme oxygenase | PA0672 | 2.809711456 |
| Alkyl hydroperoxide reductase (AhpD) | PA2331 | 2.81463623 |
| Transcriptional regulator (Dnr) | PA0527 | 2.814872106 |
| Uncharacterized protein | PA1665 | 2.881585439 |
| Alkanesulfonate monooxygenase | PA3444 | 2.959523519 |
| Uncharacterized protein | PA2033 | 3.003330866 |
| Probable chemotaxis transducer | PA1646 | 3.181678136 |
| Probable periplasmic taurine-binding protein | PA3938 | 3.245528539 |
| Uncharacterized protein | PA4129 | 3.273066839 |
| Uncharacterized protein | PA2453 | 3.4348526 |
| Aspartate 1-decarboxylase | PA4731 | 3.467517853 |
| Second ferric pyoverdine receptor (FpvB) | PA4168 | 3.497207006 |
| Uncharacterized protein | PA5346 | 3.578495026 |
| Uncharacterized protein | PA1888 | 3.58943367 |
| Probable nitroreductase | PA5190 | 3.604694366 |
| Fumarate hydratase class II | PA4470 | 3.621372223 |
| Uncharacterized protein | PA1657 | 3.901093801 |
| Cytochrome c oxidase subunit (Cbb3-type) | PA4133 | 3.952099482 |
| Uncharacterized protein | PA3445 | 4.076587041 |
| Polyphosphate:ADP/GDP phosphotransferase | PA0141 | 4.09273084 |
| Probable hemin degrading factor | PA4709 | 4.898995717 |
| Probable ClpA/B-type protease | PA1662 | 5.367067973 |
| Superoxide dismutase [Mn] | PA4468 | 5.989256541 |
| Major exported protein (Secreted protein hcp) | PA5267 | 6.057260513 |
| Uncharacterized protein | PA1658 | 6.723980586 |
