# Supplementary material for: Phosphorus stress induces the synthesis of novel glycolipids in Pseudomonas aeruginosa that confer protection against a last-resort antibiotic
Source: ISME J. 2021 May 24;15(11):3303–14. doi: 10.1038/s41396-021-01008-7 (PMC8528852; doi:10.1038/s41396-021-01008-7)
Supplement: Supplementary file 3 — supplementary table 2 [file 41396_2021_1008_MOESM3_ESM.docx]

**Supplementary table 2** Protein BLAST identification of locus tags homologous to *agt1* and *agt2* glycolipid synthesis genes in all genome sequenced *P. aeruginosa* strains at the JGI IMG database. Amino acid sequences of PA3218 (*agt1*) and PA0842 (*agt2*) from strain PAO1 were used as query sequences. Only genomes with the sequencing status labelled as “finished” are used.

| ***P. aeruginosa* strain** | ***agt2* locus tag** | ***agt1* locus tag** |
| --- | --- | --- |
| 12-4-4(59) | Ga0123778_114449 | Ga0123778_111868 |
| 14649 | Ga0071081_1121156 | Ga0071081_1151594 |
| 14650 | Ga0071078_1011198 | Ga0071078_112549 |
| 19BR | Ga0248322_11878 | Ga0248322_113734 |
| 213BR | Ga0036904_11879 | Ga0036904_113760 |
| 8380 | Ga0123667_114557 | Ga0123667_111892 |
| AES-1R | Ga0105935_112216 | Ga0105935_113158 |
| AJ D 2 | Ga0395468_854 | Ga0395468_3353 |
| AR_0354 | Ga0272040_01_2807356_2808576 | Ga0272040_01_6550596_6551651 |
| AR_0360 | Ga0272319_01_3455297_3456517 | Ga0272319_01_679654_680709 |
| AR_455 | Ga0349608_01_6011993_6013213 | Ga0349608_01_3448498_3449553 |
| AR_458 | Ga0350086_01_1364223_1365443 | Ga0350086_01_5098419_5099474 |
| AR441 | Ga0349683_01_4672877_4674097 | Ga0349683_01_1879124_1880179 |
| ATCC 15692 | Ga0174979_114286 | Ga0174979_111794 |
| ATCC 27853 | Ga0133450_114562 | Ga0133450_111789 |
| B10W | Ga0174977_114731 | Ga0174977_111880 |
| B136-33 | G655_21145 | G655_08740 |
| BA7823 | Ga0399483_01_4564387_4564986 | Ga0399483_01_1737804_1738859 |
| BAMCPA07-48 | Ga0133421_114930 | Ga0133421_111958 |
| c7447m | Ga0034254_00877 | Ga0034254_03392 |
| Carb01 63 | Ga0123806_115199 | Ga0123806_112201 |
| Cu1510 | Ga0125155_112051 | Ga0125155_114535 |
| DHS01 | DPADHS01_04365 | DPADHS01_19380 |
| DK2 | PADK2_21495 | PADK2_08235 |
| DSM 50071 | Ga0081678_114289 | Ga0081678_111769 |
| F22031 | Ga0133363_114486 | Ga0133363_111866 |
| F23197 | Ga0123716_114372 | Ga0123716_111775 |
| F30658 | Ga0123814_114858 | Ga0123814_111922 |
| F5677 | Ga0272182_01_4774431_4775651 | Ga0272182_01_2008248_2009303 |
| F63912 | Ga0125144_114612 | Ga0125144_111815 |
| F9670 | Ga0123675_114554 | Ga0123675_111797 |
| F9676 | Ga0081746_114350 | Ga0081746_111812 |
| H27930 | Ga0123748_114400 | Ga0123748_111813 |
| H47921 | Ga0123745_114788 | Ga0123745_111916 |
| H5708 | Ga0125197_114357 | Ga0125197_111787 |
| IOMTU 133 | Ga0125117_114705 | Ga0125117_111964 |
| LES400 | T222_23200 | T222_09615 |
| LES431 | T223_22870 | T223_09285 |
| LESB65 | T224_22800 | T224_09600 |
| M1608 | Ga0125123_111780 | Ga0125123_114161 |
| M18 | PAM18_4198 | PAM18_1753 |
| M37351 | Ga0123746_111779 | Ga0123746_114606 |
| MTB-1 | U769_21605 | U769_08820 |
| N17-1 | Ga0133283_114431 | Ga0133283_111775 |
| NCGM 1984 | Ga0069487_114712 | Ga0069487_111829 |
| NCGM1900 | Ga0069483_111893 | Ga0069483_113106 |
| NCGM2.S1 | NCGM2_1616 | NCGM2_4333 |
| NCGM257 | Ga0123816_114842 | Ga0123816_111998 |
| NCTC10332 | Ga0111760_114317 | Ga0111760_111779 |
| NHmuc | Ga0174978_111296 | Ga0174978_113770 |
| PA_150577 | Ga0258935_01_4647317_4648537 | Ga0258935_01_1875598_1876653 |
| PA_D1 | Ga0175649_114525 | Ga0175649_111883 |
| PA_D16 | Ga0175650_114559 | Ga0175650_111883 |
| PA_D2 | Ga0175975_114523 | Ga0175975_111883 |
| PA_D21 | Ga0174817_114522 | Ga0174817_111884 |
| PA_D22 | Ga0175478_114563 | Ga0175478_111883 |
| PA_D25 | Ga0175479_114572 | Ga0175479_111886 |
| PA_D5 | Ga0175314_114558 | Ga0175314_111883 |
| PA_D9 | Ga0175801_114530 | Ga0175801_112426 |
| PA1 | PA1S_gp4364 | PA1S_gp1049 |
| PA1088 | Ga0174816_114598 | Ga0174816_111775 |
| PA11803 | Ga0175651_115032 | Ga0175651_112016 |
| PA121617 | Ga0175312_114401 | Ga0175312_111813 |
| PA1R | PA1R_gp4364 | PA1R_gp1049 |
| PA1RG | Ga0113987_114499 | Ga0113987_111869 |
| PA7790 | Ga0174976_124813 | Ga0174976_121825 |
| PA8281 | Ga0175803_114734 | Ga0175803_111816 |
| PA83 | Ga0226249_01_5016223_5017443 | Ga0226249_01_1967397_1968452 |
| PA96 | PA96_4322 | PA96_1712 |
| PACS2 | PaerPA_01001324 | PaerPA_01003815 |
| PAK | Y880_0122405 | Y880_0104770 |
| **PAO1** | **PA0842** | **Ga0097782_15205/PA3218** |
| PAO581 | Ga0034253_00876 | Ga0034253_03217 |
| PSE305 | Ga0098285_111610 | Ga0098285_114354 |
| RP73 | M062_04095 | M062_17180 |
| S04 90 | Ga0175152_124808 | Ga0175152_121916 |
| S86968 | Ga0125010_114699 | Ga0125010_111890 |
| SCV20265 | SCV20265_4686 | SCV20265_1838 |
| SCVFeb | Ga0175802_114242 | Ga0175802_111766 |
| SCVJan | Ga0175480_114242 | Ga0175480_111766 |
| SJTD-1 | A214_02884 | A214_05412 |
| ST277 | Ga0062142_101948 | Ga0062142_101386 |
| sv. O12 PA7 | PSPA7_4678 | PSPA7_1909 |
| T38079 | Ga0123648_114521 | Ga0123648_111780 |
| T52373 | Ga0125064_114327 | Ga0125064_111765 |
| T63266 | Ga0123787_114538 | Ga0123787_111765 |
| UCBPP-PA14 | PA14_53380 | PA14_22600 |
| VA-134 | Ga0133270_114297 | Ga0133270_111758 |
| W16407 | Ga0125011_114694 | Ga0125011_111935 |
| W36662 | Ga0125121_114683 | Ga0125121_111960 |
| W45909 | Ga0125012_114687 | Ga0125012_111860 |
| W60856 | Ga0123715_111730 | Ga0123715_112390 |
| X78812 | Ga0123617_114291 | Ga0123617_111776 |
| YL84 | AI22_11705 | AI22_24595 |
|  |  |  |
