# Supplementary material for: Phosphorus stress induces the synthesis of novel glycolipids in Pseudomonas aeruginosa that confer protection against a last-resort antibiotic
Source: ISME J. 2021 May 24;15(11):3303–14. doi: 10.1038/s41396-021-01008-7 (PMC8528852; doi:10.1038/s41396-021-01008-7)
Supplement: Supplementary file 4 — supplementary table 3 [file 41396_2021_1008_MOESM4_ESM.docx]

**Supplementary table 3**. Proteomic analysis of wild type *P. aeruginosa* PAO1 versus the Δ*plcP* mutant grown at 50 µM phosphate. Locus tags from the PAO1 genome reference database are listed alongside known or predicted protein functions. Log2 transformation of fold change values show the differences between WT *P. aeruginosa* and glycolipid deficient Δ*plcP*, and are the mean of 3 replicates. All glycolipid responsive proteins displayed have a log2(Fold Change) of ≥2 and all were considered significant with a false detection rate (FDR) of <0.05. Proteins that are more represented in the mutant are indicated with negative values.

| **Gene locus** | **Log2(Fold Change)** | **FDR** | **Protein** | **Function** |
| --- | --- | --- | --- | --- |
| PA4686 | -3.6 | 0.006 | MksF | Condensin |
| PA2491 | -6.1 | 0.000 | MexS | Negative regulator of type III secretion system |
| PA4500 | -2.5 | 0.001 | DppA3 | Solute binding protein specific for dipeptides |
| PA2483 | -5.3 | 0.002 | - | unknown |
| PA4582 | -3.4 | 0.004 | - | unknown |
| PA4502 | -2.3 | 0.000 | DppA4 | Solute binding protein specific for tripeptides |
| PA2462 | -3.9 | 0.002 | - | unknown |
| PA2476 | -2.2 | 0.025 | DsbG | Thiol:disulfide interchange protein |
| PA2464 | -1.9 | 0.013 | - | unknown |
| PA5190 | 3.1 | 0.019 | - | unknown |
| PA4527 | 4.2 | 0.000 | PilC | Inner membrane core protein in type IV pili |
| PA4468 | 5.7 | 0.000 | SodM | Superoxide dismutase |
| PA4221 | 5.1 | 0.000 | FptA | Fe(III)-pyochelin outer membrane receptor |
